# Workplace factors associated with job satisfaction among dental hygienists and assistants in the United States

**DOI:** 10.1093/haschl/qxae147

**Published:** 2025-01-13

**Authors:** Nozomi Sasaki, Jinman Pang, Simona Surdu, Rachel W Morrissey, Marko Vujicic, Jean Moore

**Affiliations:** Oral Health Workforce Research Center, Center for Health Workforce Studies, College of Integrated Health Sciences, University at Albany, State University of New York, Rensselaer, NY 12144, United States; Oral Health Workforce Research Center, Center for Health Workforce Studies, College of Integrated Health Sciences, University at Albany, State University of New York, Rensselaer, NY 12144, United States; Oral Health Workforce Research Center, Center for Health Workforce Studies, College of Integrated Health Sciences, University at Albany, State University of New York, Rensselaer, NY 12144, United States; ADA Health Policy Institute, Chicago, IL 60611, United States; ADA Health Policy Institute, Chicago, IL 60611, United States; Oral Health Workforce Research Center, Center for Health Workforce Studies, College of Integrated Health Sciences, University at Albany, State University of New York, Rensselaer, NY 12144, United States

**Keywords:** job satisfaction, workplace factors, dental hygienists, dental assistants

## Abstract

Previous research has assessed job satisfaction and related workplace factors among healthcare workers. However, studies on dental care professionals are limited. This study aimed to evaluate job satisfaction among US dental hygienists (DHs) and assistants (DAs) and identify workplace factors contributing to their job satisfaction or dissatisfaction. A cross-sectional study was conducted using survey data collected from DHs (*n* = 4078) and DAs (*n* = 2945) actively working in 2022. Descriptive statistics and multivariable logistic regressions were used to assess associations between workplace factors and job satisfaction, adjusting for demographics, practice patterns, and geography. Over 80% of DHs and DAs reported being satisfied with their jobs. Positive workplace culture, practice philosophy, opportunities for growth and advancement, good communication in practice, work-life balance, fair pay, a reasonable workload, and flexible work schedules are associated with job satisfaction. Overwork was associated with job dissatisfaction among DAs. Most DHs and DAs were satisfied with their current jobs, with positive workplace culture, growth opportunities, and effective communication being key factors contributing to their satisfaction. This study highlights the importance of developing strategies to improve workplace environments and promote the well-being and retention of oral health providers.

## Introduction

In the United States, the dental workforce is experiencing a notable downturn in the number of dental hygienists (DHs) and dental assistants (DAs). This decline, identified as a 10% decrease in practice capacity in 2022, affects the efficiency and quality of dental care services and poses significant challenges to the well-being of the oral health workforce.^1^ Moreover, the COVID-19 pandemic exacerbated this decline by intensifying oral healthcare workers' intentions to leave the profession and accelerating retirement due to increasing anxiety about the risk of virus transmission during dental procedures.^2,3^ The oral health workforce has not yet returned to prepandemic levels.^4^ Previous studies have shown that work environment, job satisfaction, and burnout are associated with intention to leave and employee turnover.^1,5,6^ These studies suggest critical considerations for maintaining dental service delivery and supporting the well-being of these essential oral health professionals.^1-3,5^

In the broader healthcare system, job satisfaction among healthcare workers (HCWs) is well studied and has been shown to affect employee well-being and patient care outcomes. Higher job satisfaction among HCWs is associated with lower levels of burnout, reduced turnover intentions, and improved quality of care.^7,8^ Conversely, job dissatisfaction can lead to significant negative impacts on health care due to increased absenteeism and staff turnover and diminished patient satisfaction and safety.^9,10^ Several studies have emphasized that HCWs in better work environments show significantly lower job dissatisfaction and burnout rates and achieve a better quality of care.^9,11^ However, the roles of oral health professionals and HCWs differ regarding work tasks, professional training, patient interactions, work environments, and specific stressors. Therefore, addressing the specific workplace factors influencing job satisfaction among DHs and DAs is essential, as they play a vital role in the dental care delivery system.

In dental practice, job satisfaction affects the personal and professional lives of DHs and DAs and may impact patients' experiences and outcomes. Among US DHs, higher job satisfaction is negatively associated with burnout.^6,12^ In contrast, DHs who consider departing from the profession within 5 years exhibit significantly lower satisfaction and greater fatigue and burnout.^13^ Although a few studies have identified contributing factors to job dissatisfaction among oral health professionals, such as overwork, emotional demands, stress, and income,^12,14^ our understanding of workplace factors influencing job satisfaction among DHs and DAs remains limited. Furthermore, many studies have focused primarily on dentist burnout or job satisfaction,^5,14-19^ whereas only a few studies have examined job satisfaction and associated factors among DHs, and even fewer have addressed DAs. Most of these studies were conducted outside of the United States, where oral health delivery systems may differ.^20-25^ In addition, prior US studies have often relied on research conducted using outdated data, convenience samples, or narrow scopes focused on occupational stress and burnout, limiting their relevance to the challenges DHs and DAs currently face. In our study, we aimed to address these knowledge gaps by evaluating job satisfaction among DHs and DAs in the United States and identifying contributing workplace factors using recent online survey data collected by the American Dental Association (ADA) in 2022. This dataset is unique in its comprehensive coverage of job satisfaction and workplace factors among DHs and DAs at a national level, providing insights that are not available in other datasets. Our study provides a deeper understanding of workplace dynamics within the oral health workforce and valuable insights that can support the well-being of oral health professionals, improve the quality of patient care, and promote the efficient operation of dental practices.

## Materials and methods

### Data collection

This cross-sectional study used survey data collected in June 2022 by the ADA, Health Policy Institute (HPI), American Dental Hygienists' Association (ADHA), American Dental Assistants Association (ADAA), Dental Assisting National Board (DANB), and IgniteDA. The survey was distributed to current or past DHs and DAs. Approximately 124 000 survey invitations were emailed to DHs and 133 000 to DAs. The anonymous survey was administered online in June 2022 via the Qualtrics Core XM (Qualtrics, Provo, UT) platform and included questions on work characteristics, job satisfaction, and potential workplace factors contributing to job satisfaction or dissatisfaction. A total of 5122 DHs and 4225 DAs participated in this survey, yielding an unadjusted response rate of approximately 4% among DHs. The response rate among DHs is an approximation, given the uncertainty regarding the presence of duplicate emails or invalid addresses that may have prevented the survey from reaching the intended recipients. The contact email lists used by the dental assisting organizations were not mutually exclusive; therefore, the DA response rate could not be calculated. The project was deemed exempt from review by the ADA Institutional Review Board. More details about the data collection methods and survey content can be found in a technical report.^1^

### Measurement of job satisfaction and potential workplace factors contributing to job satisfaction or dissatisfaction

This study employed a single-item measure with a 10-point scale to assess job satisfaction levels: “On a scale of 1-10, how satisfied are you in your current role? (1 = not at all satisfied, 10 = extremely satisfied)”. This 10-point scale is relatively simple and captures respondents' variations in overall job satisfaction, with satisfactory validity and reliability compared with multiple-item or 4-point scale measures.^26-28^ Dental hygienists and DAs who indicated high levels of job satisfaction (scores from 6-10) were asked what contributed to their satisfaction, whereas those who indicated low levels of job satisfaction (scores from 1-5) were asked what contributed to their dissatisfaction: “You indicated that you are at least somewhat satisfied [dissatisfied] in your current role. What are the top 3 reasons why?”. A list of 11 workplace factors was provided to both DH and DA survey respondents who were satisfied and those who were dissatisfied with their current job, including pay, benefits, work-life balance, the opportunity for growth and advancement, workload, workplace culture (eg, leadership style, values, and coworkers), communication in practice, work schedules, practice philosophy, patients, and safety (eg, COVID-19 protocols). Survey respondents who reported satisfaction with their jobs had an additional potential contributing factor, “helping patients improve oral health”.

### Measurement of covariates

Potential confounding variables were identified according to associations suggested in the literature.^1,2^ These variables included age (under 35, 35-44, 45-54, and 55 and over), race and ethnicity (non-Hispanic White, non-Hispanic non-White, and Hispanic), employment status (full-time or part-time), tenure (less than 1 year, 1-2 years, 3-5 years, 6-10 years, 11-20 years, and more than 20 years), primary practice setting (private solo practice, group, specialty practice/multispecies clinic, dental service organization [DSO], public health/federally qualified health center [FQHC]/community health center [CHC], and academic settings), location based on the rural-urban commuting area (metropolitan, micropolitan, and small town), and geographic region (Northeast, Midwest, South, and West).

### Statistical analysis

Survey participants who were employed in a different occupation in or outside dentistry, were semiretired/performed limited work, or were fully retired were excluded, resulting in final study samples of 4078 DHs and 2945 DAs. Descriptive analyses using the Wald chi-square test were employed to evaluate differences in respondents' job satisfaction according to their characteristics. Multivariable logistic regressions were used to assess the associations between job satisfaction (very satisfied [scores from 8-10] vs somewhat satisfied [scores from 6-7]), job dissatisfaction (very dissatisfied [scores from 1-3] vs somewhat dissatisfied [scores from 4-5]), workplace factors, demographics, and work characteristics for each oral health profession separately by estimating odds ratios (ORs) and 95% CIs. All analyses were conducted using SAS software version 9.4 (SAS Institute Inc., Cary, NC) and STATA version 17.0 (Stata Corp., College Station, TX). The significance level was set at 0.05 for all the statistical tests.

## Results

The demographic characteristics of the study participants are shown in Table 1. Over 80% of DHs and DAs were satisfied with their jobs, whereas approximately 20% were dissatisfied. On average, a greater proportion of DHs who were satisfied with their current job compared to those dissatisfied were under 35 years of age (27.6% vs 23.6%), employed full-time (65.9% vs 61.3%), and worked in dental practices located in the West region (30.6% vs 23.1%). In contrast, proportionally more DHs working in DSOs were dissatisfied with their job (14.2% vs 8.0%). On average, compared with those who were dissatisfied with their current job, a greater proportion of DAs who were satisfied with their current job were non-Hispanic White (71.2% vs 65.3%), reported 6-10 years of tenure (12.9% vs 10.0%), worked in private solo practices (40.4% vs 35.1%), and practiced in the Midwest (31.9% vs 23.8%) or West (19.2% vs 17.5%) regions.

**Table 1.** Characteristics of dental hygienists and assistants by job satisfaction, 2022.

Variables of interest	Job satisfaction of dental hygienists			Job satisfaction of dental assistants		
	Satisfied (*n* = 3275)	Dissatisfied (*n* = 803)		Satisfied (*n* = 2391)	Dissatisfied (*n* = 554)	
	*n* (%)	*n* (%)	*P*-value	*n* (%)	*n* (%)	*P*-value
Gender	**3120**	**748**	**<0**.**001**	**2194**	**505**	**0**.**760**
Male	46 (1.5)	15 (2.0)		59 (2.7)	13 (2.6)	
Female	3057 (98.0)	718 (96.0)		2109 (96.1)	484 (95.8)	
Others	17 (0.5)	15 (2.0)		26 (1.2)	8 (1.6)	
Age (years)	**3064**	**713**	**<0**.**001**	**2133**	**482**	**0**.**220**
Under 35	845 (27.6)	168 (23.6)		759 (35.6)	171 (35.5)	
35-44	808 (26.4)	229 (32.12)		428 (20.1)	116 (24.1)	
45-54	674 (22.0)	180 (25.3)		435 (20.4)	99 (20.5)	
55 and over	737 (24.0)	136 (19.1)		511 (23.9)	96 (19.9)	
Race/ethnicity	**3067**	**738**	**0**.**320**	**2133**	**490**	**0**.**022**
Non-Hispanic, White	2440 (79.6)	574 (77.8)		1519 (71.2)	320 (65.3)	
Non-Hispanic, non-White^a^	308 (10.0)	88 (11.9)		314 (14.7)	94 (19.2)	
Hispanic	319 (10.4)	76 (10.3)		300 (14.1)	76 (15.5)	
Employment status	**3275**	**803**	**0**.**013**	**2391**	**554**	**0**.**240**
Full-time	2159 (65.9)	492 (61.3)		2062 (86.2)	467 (84.3)	
Part-time	1116 (34.1)	311 (38.7)		329 (13.8)	87 (15.7)	
Tenure	**3217**	**782**	**0**.**260**	**2309**	**532**	**0**.**003**
Less than 1 year	378 (11.7)	81 (10.4)		332 (14.4)	83 (15.6)	
1-2 years	922 (28.7)	243 (31.1)		661 (28.6)	183 (34.4)	
3-5 years	661 (20.5)	169 (21.6)		426 (18.4)	105 (19.7)	
6-10 years	446 (13.9)	116 (14.8)		299 (12.9)	53 (10.0)	
11-20 years	484 (15.0)	111 (14.2)		275 (11.9)	63 (11.8)	
More than 20 years	326 (10.2)	62 (7.9)		316 (13.7)	45 (8.5)	
Primary practice setting	**3224**	**791**	**<0**.**001**	**2333**	**542**	**0**.**043**
Private solo	1628 (50.5)	397 (50.2)		942 (40.4)	190 (35.1)	
Group	793 (24.6)	181 (22.9)		523 (22.4)	126 (23.2)	
Specialty practice/multi specialty clinic	159 (4.9)	27 (3.4)		332 (14.2)	80 (14.8)	
DSO	259 (8.0)	112 (14.2)		148 (6.3)	52 (9.6)	
Public health/FQHCs/CHCs	191 (5.9)	40 (5.1)		190 (8.1)	40 (7.4)	
Academic^b^	194 (6.0)	34 (4.3)		198 (8.5)	54 (10.0)	
Primary practice location	**3015**	**706**	**0**.**230**	**2096**	**474**	**0**.**410**
Metropolitan	2622 (87.0)	622 (88.1)		1752 (83.6)	406 (85.6)	
Micropolitan	236 (7.8)	58 (8.2)		205 (9.8)	44 (9.3)	
Small town/rural	157 (5.2)	26 (3.7)		139 (6.6)	24 (5.1)	
Geographic region^c^	**3015**	**706**	**0**.**001**	**2096**	**474**	**0**.**001**
Northeast	471 (15.6)	134 (19.0)		568 (27.1)	156 (32.9)	
Midwest	733 (24.3)	182 (25.8)		669 (31.9)	113 (23.8)	
South	889 (29.5)	227 (32.1)		456 (21.8)	122 (25.7)	
West	922 (30.6)	163 (23.1)		403 (19.2)	83 (17.5)	

The satisfied group included respondents who rated job satisfaction between 6 and 10 points; the dissatisfied group consisted of respondents who rated job satisfaction between 1 and 5 points on a 10-point scale. The difference between the satisfied and dissatisfied groups was determined by the Wald χ^2^ test; *P* < 0.05 was considered significant. Bold values indicate the total number of observations for each variable within each provider and job satisfaction group, along with the significance level for differences. Source: ADA Health Policy Institute. Dental Hygienists and Dental Assistants Workforce Shortage Survey, 2022.

Abbreviations: DSO, dental service organization; FQHCs, federally qualified health centers; CHCs, community health centers.

^a^Non-Hispanic, non-White includes Black, American Indian, Alaskan Native, Asian, Native Hawaiian, and Pacific Islander.

^b^Academic settings include university, college, school-based, military, independent, and other.

^c^The Northeast region includes Connecticut, Maine, Massachusetts, New Hampshire, New Jersey, New York, Pennsylvania, Rhode Island, and Vermont. The Midwest region encompasses Illinois, Indiana, Iowa, Kansas, Michigan, Minnesota, Missouri, Nebraska, North Dakota, Ohio, South Dakota, and Wisconsin. The South region comprises Alabama, Arkansas, Delaware, the District of Columbia, Florida, Georgia, Kentucky, Maryland, Mississippi, North Carolina, Oklahoma, South Carolina, Texas, Virginia, and West Virginia. The West region comprises Alaska, Arizona, California, Colorado, Hawaii, Idaho, Montana, Nevada, New Mexico, Oregon, Utah, Washington, and Wyoming.

**Figure 1.**
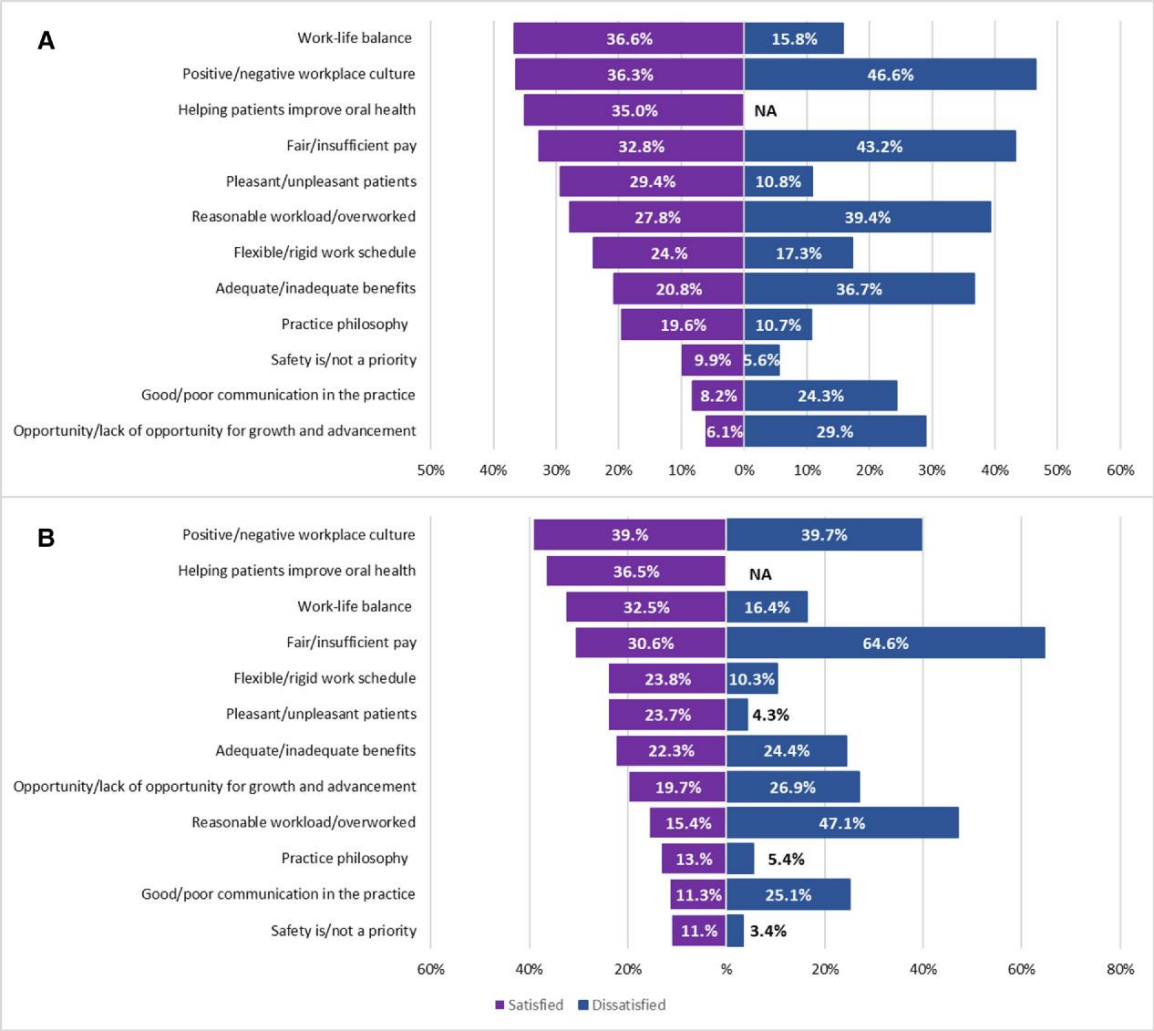
Workplace factors contributing to job satisfaction and dissatisfaction among dental hygienists and dental assistants, 2022. Source: ADA Health Policy Institute. Dental Hygienists and Dental Assistants Workforce Shortage Survey, 2022. The satisfied group included respondents who rated job satisfaction between 6 and 10 points; the dissatisfied group consisted of respondents who rated job satisfaction between 1 and 5 points on a 10-point scale. Survey respondents who reported job satisfaction had the option to identify an additional contributing factor: “helping patients improve oral health.” (A) Dental hygienists. (B) Dental assistants.

Figure 1A and B shows the most common workplace factors contributing to job satisfaction and dissatisfaction among DHs and DAs. Dental hygienists reported the following top reasons for their job satisfaction: positive work-life balance (36.6%), positive workplace culture (36.3%), ability to help patients improve oral health (35.0%), and fair pay (32.8%). In contrast, the main reasons for dissatisfaction among DHs were negative workplace culture (46.6%), insufficient pay (43.2%), overwork (39.4%), and inadequate benefits (36.7%). Similarly, DAs reported the following top reasons for their job satisfaction: positive workplace culture (39.0%), ability to help patients improve oral health (36.5%), work-life balance (32.5%), and fair pay (30.6%). The main reasons for dissatisfaction among DAs were insufficient pay (64.6%), overwork (47.1%), and negative workplace culture (39.7%).

**Figure 2.**
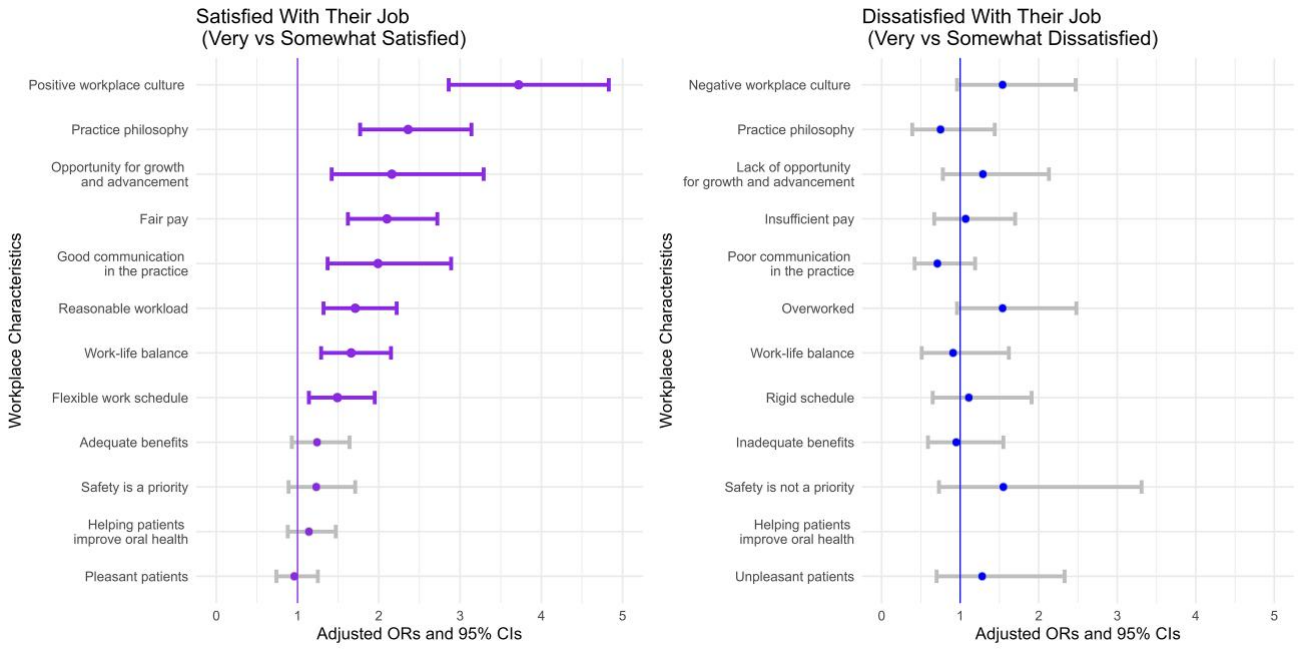
Adjusted associations between workplace factors and job satisfaction among dental hygienists, 2022. Source: ADA Health Policy Institute. Dental Hygienists and Dental Assistants Workforce Shortage Survey, 2022. The satisfied group included those who rated it between 6 and 10 (very satisfied, ranging from 8-10; somewhat satisfied, ranging from 6-7); the dissatisfied group consisted of respondents who rated job satisfaction between 1 and 5 (very dissatisfied, ranging from 1-3; somewhat dissatisfied, ranging from 4-5) on a 10-point scale. Survey respondents who reported job satisfaction had the option to identify an additional contributing factor: “helping patients improve oral health.” Multivariable logistic regression estimates (odds ratio [OR] and 95% CI) were adjusted for age, race and ethnicity, employment status, tenure, primary practice setting, urban/rural location, and geographic region.

Figure 2 and Table S1 show the estimated ORs and 95% CIs from multivariable logistic regression analyses among DHs. Most DHs reported high job satisfaction, with 54.3% very satisfied and 26.0% somewhat satisfied, whereas 19.7% expressed dissatisfaction with their job (7.6% very dissatisfied; 12.1% somewhat dissatisfied [results not shown]). Adjusted regression estimates found significant associations of DHs' job satisfaction with positive workplace culture (OR = 3.72, 95% CI = 2.86-4.83), practice philosophy (OR = 2.36, 95% CI = 1.77-3.14), opportunities for growth and advancement (OR = 2.16, 95% CI = 1.42-3.29), fair pay (OR = 2.10, 95% CI = 1.62-2.72), effective communication in practice (OR = 1.99, 95% CI = 1.37-2.89), reasonable workload (OR = 1.71, 95% CI = 1.32-2.22), good work-life balance (OR = 1.66, 95% CI = 1.29-2.15), and flexible work schedules (OR = 1.49, 95% CI = 1.14-1.95). No significant associations were found between job dissatisfaction and workplace factors.

**Figure 3.**
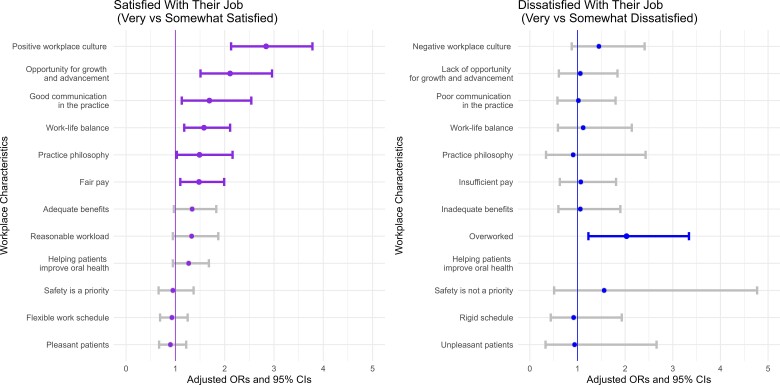
Adjusted associations between workplace factors and job satisfaction among dental assistants, 2022. Source: ADA Health Policy Institute. Dental Hygienists and Dental Assistants Workforce Shortage Survey, 2022. The satisfied group included those who rated it between 6 and 10 (very satisfied, ranging from 8-10; somewhat satisfied, ranging from 6-7); the dissatisfied group consisted of respondents who rated job satisfaction between 1 and 5 (very dissatisfied, ranging from 1-3; somewhat dissatisfied, ranging from 4-5) on a 10-point scale. Survey respondents who reported job satisfaction had the option to identify an additional contributing factor: “helping patients improve oral health.” Multivariable logistic regression estimates (odds ratio [OR] and 95% CI) were adjusted for age, race and ethnicity, employment status, tenure, primary practice setting, urban/rural location, and geographic region.

Figure 3 and Table S1 show the estimated ORs with 95% CIs from multivariable logistic regression analyses among DAs. Most DAs reported high job satisfaction, with 59.6% very satisfied and 21.6% somewhat satisfied, whereas 18.8% expressed dissatisfaction with their job (7.2% very dissatisfied; 11.6% somewhat dissatisfied [results not shown]). Adjusted regression estimates indicated that DAs' job satisfaction was significantly associated with positive workplace culture (OR = 2.84, 95% CI = 2.13-3.78), opportunities for growth and advancement (OR = 2.11, 95% CI = 1.51-2.96), good communication in practice (OR = 1.69, 95% CI = 1.13-2.54), work-life balance (OR = 1.58, 95% CI = 1.18-2.11), practice philosophy (OR = 1.49, 95% CI = 1.03-2.16), and fair pay (OR = 1.48, 95% CI = 1.10-1.99). Dental assistants' job dissatisfaction was significantly associated with overwork (OR = 2.03, 95% CI = 1.23-3.34).

## Discussion

This study revealed that most DHs and DAs were satisfied with their jobs. The key factors contributing to job satisfaction included a positive workplace culture, practice philosophy, opportunities for growth and advancement, effective communication in the dental practice, good work-life balance, and fair pay.

The results indicated that over 80% of DHs and DAs were satisfied with their jobs, with approximately 54% of DHs and 60% of DAs reporting high satisfaction levels. These findings are comparable with those of a meta-analysis of dentists' job satisfaction, which showed 76.6% were satisfied with their jobs.^14^ Research among other healthcare providers has also indicated comparable job satisfaction in the US nursing workforce, with 60% of registered nurses reporting very high or high job satisfaction and 29% reporting moderate job satisfaction.^29^

Job satisfaction has long been recognized as a key determinant of staff retention, quality of care, and a supportive workplace environment.^19,30-32^ Understanding job satisfaction and its complex relationships with the work environment, compensation, psychological and physiological conditions, and safety often involves applying specific theories from healthcare psychology. Two theories commonly utilized in research are Maslow's hierarchy of needs and Herzberg's motivator-hygiene theory.^33-35^ Maslow's hierarchy of needs divides human needs into 5 components: fundamental physiological needs, security and safety needs, belonging needs, esteem needs, and self-actualization needs.^33,35^ These needs motivate the workforce in a hierarchical order.^33,35^ In contrast, Herzberg's motivator-hygiene theory classifies job environment factors into intrinsic motivators supporting job satisfaction and extrinsic hygiene factors that prevent dissatisfaction.^35^ Despite the application of these psychological theories, job satisfaction among the dental workforce is still not well understood and requires further research.

International and US studies have identified key workplace factors associated with job satisfaction among DHs and DAs, although their specific roles may vary according to each country's policy framework. Despite these distinctions, it is evident that similar workplace factors influence job satisfaction. For example, a qualitative study of active DHs in Sweden emphasized that positive coworker relationships, visible work results, and autonomy in decision-making were significant motivating factors,^21^ whereas a study in Japan reported that job satisfaction was more closely linked to factors such as contributing to community welfare, protecting patients' health, finding their work fulfilling, and the need for high levels of specialty and skills than to employment benefits.^20^ Earlier research in the United States identified significant determinants of job satisfaction among DHs, including salary, supervisory style, work-life balance, recognition, and engagement in practice.^30^ A more recent US study reported that DHs with greater job satisfaction were less likely to consider leaving their positions, while increased levels of disengagement and exhaustion in practice were associated with a greater intention to leave.^6^ These studies support our findings. Additionally, our findings extend those of previous studies, showing that flexible work schedules and opportunities for growth and advancement are also significant factors influencing DHs' job satisfaction.

Our study results regarding job satisfaction and associated workplace factors among DAs are consistent with those of previous studies conducted mostly outside the United States. Canadian studies have identified work-related fatigue, time pressure, nonpatient tasks, low income, limited opportunities for professional development, and a lack of recognition as contributors to increased work-related stress, emotional burden, and job dissatisfaction.^22,23^ Similarly, a study in Brazil found that low job satisfaction among DAs was influenced by low salaries, dental clinic practice settings, overwork, and dentist personalities.^24^ A study in Saudi Arabia highlighted that work-life balance, quality of service, salary, prestige, and self-respect were important job satisfaction factors among DAs.^25^ Our study results also identified effective communication in practice and positive workplace culture as key workplace factors associated with job satisfaction among US DAs. Importantly, our study confirmed that being overworked leads to job dissatisfaction. These findings expand the limited US studies evaluating the significant impact of workplace factors on job satisfaction among oral health professionals.

Several strategies have been suggested to increase job satisfaction among HCWs, but the current literature does not identify effective interventions specifically for oral health professionals. A study showed that training in communication and management skills can reduce work-related stress and mental health issues.^36^ Additionally, workshops that enhances communication and balance professional roles with personal needs can reduce defensive reactions to depersonalization and improve perceptions of personal accomplishment.^37^ Another study found that organizational interventions focusing on effective communication and workflow optimizations improved clinician satisfaction and reduced turnover intentions.^38^ Mindfulness-based stress reduction programs were also effective in reducing emotional exhaustion, depersonalization, depression, anxiety, and stress, while enhancing feelings of personal accomplishment.^39^ These interventions highlight the important roles of organizational and personal development as potential strategies for enhancing job satisfaction among DHs and DAs. Dental practices can implement structural changes that support work-life balance and improve communication to ensure a positive work culture for DHs and DAs.

This study has several limitations and strengths due to its design and methodology. First, the cross-sectional nature of our study, with data collected at a single point in time, limits our ability to establish causal relationships. Second, although our statistical models accounted for regional variations, differences in responsibilities permitted under state practice laws may influence the outcomes. Third, our sample size was not large due to a low response rate, and we could not extend our findings to the broader population of oral health professionals. Nonetheless, our study is the first to incorporate a wide range of workplace characteristics and provide timely insights into the factors influencing job satisfaction among oral health professionals, especially during ongoing workforce shortages exacerbated by the COVID-19 pandemic. These findings underscore the importance of identifying strategies to improve the workplace environment and promote the well-being and retention of DHs and DAs. A better understanding of various work-related factors influencing job satisfaction can guide efforts to reduce attrition, turnover, and shortages in the oral health workforce. Job satisfaction among DHs and DAs plays an important role in staff retention and the quality of care.^6,12,13^ Our study also highlights challenges contributing to job dissatisfaction, including being overworked. Potential interventions to improve job satisfaction may include both organizational and individual approaches to enhance the management of work schedules, support positive communication and work culture, and implement mindfulness and skill development workshops. Future longitudinal studies with larger sample sizes would be valuable in enhancing our understanding of the causal relationships between identified workplace factors and job satisfaction among DHs and DAs. Additionally, further research is critical to assess burnout using a validated measure and evaluate the interconnections between workplace factors, job satisfaction, burnout, and the intention to leave to inform effective strategies for improving the work environment and retention of oral health providers.

## Conclusions

This study identified significant workplace factors associated with job satisfaction among DHs and DAs in the United States. Our findings emphasize the importance of positive workplace culture, growth opportunities, effective communication, practice philosophy, work-life balance, and fair pay in fostering high job satisfaction among DHs and DAs. By enhancing our understanding of workplace dynamics within the oral health workforce, our findings contribute to improving workplace conditions and informing policy initiatives aimed at increasing job satisfaction, retention rates, and quality of care within the US oral health sector.

## Supplementary Material

qxae147_Supplementary_Data

## Data Availability

The data in this analysis were shared through a data use agreement with the ADA Health Policy Institute. Per the data use agreement, that data are are not permitted to be shared.
