# Supplementary material for: Workplace factors associated with job satisfaction among dental hygienists and assistants in the United States
Source: Health Aff Sch. 2025 Jan 13;3(1):qxae147. doi: 10.1093/haschl/qxae147 (PMC11726826; doi:10.1093/haschl/qxae147)
Supplement: qxae147_Supplementary_Data [file qxae147_supplementary_data.zip › Supplementary_material (9.11.2024).docx]

**Supplementary material**

**Table 1.** Adjusted Odds Ratio (OR) and 95% Confidence Interval (CI) Estimates of Associations between Workplace Factors and Job Satisfaction among Dental Hygienists and Assistants, 2022

| Variables of Interest | Dental Hygienists | | Dental Assistants | |
| --- | --- | --- | --- | --- |
|  | Satisfied | Dissatisfied | Satisfied | Dissatisfied |
|  | Very satisfied (8–10) vs Somewhat satisfied (6–7) | Very dissatisfied (1–3) vs Somewhat dissatisfied (4–5) | Very satisfied (8–10) vs Somewhat satisfied (6–7) | Very dissatisfied (1–3) vs Somewhat dissatisfied (4–5) |
|  | OR (95% CI) | OR (95% CI) | OR (95% CI) | OR (95% CI) |
| **Workplace Characteristics (reference: No)** | **n = 2,947** | **n = 683** | **n = 2,020** | **n = 451** |
| Fair pay | 2.10  (1.62, 2.72)*** | 1.07  (0.67, 1.71) | 1.48  (1.10, 1.99)** | 1.07  (0.63, 1.81) |
| Adequate benefits | 1.24  (0.93, 1.64) | 0.95  (0.59, 1.55) | 1.34  (0.97, 1.83) | 1.06  (0.60, 1.90) |
| Work–life balance | 1.66  (1.29, 2.15)*** | 0.91  (0.51, 1.62) | 1.58  (1.18, 2.11)** | 1.12  (0.59, 2.15) |
| Opportunity for growth and advancement | 2.16  (1.42, 3.29)*** | 1.29  (0.78, 2.13) | 2.11  (1.51, 2.96)*** | 1.06  (0.61, 1.84) |
| Reasonable workload | 1.71  (1.32, 2.22)*** | 1.54  (0.96, 2.48) | 1.33  (0.95, 1.87) | 2.03  (1.23, 3.34)** |
| Workplace culture | 3.72  (2.86, 4.83)*** | 1.54  (0.96, 2.47) | 2.84  (2.13, 3.78)*** | 1.45  (0.88, 2.41) |
| Communication in the practice | 1.99  (1.37, 2.89)*** | 0.71  (0.42, 1.19) | 1.69  (1.13, 2.54)** | 1.02  (0.58, 1.80) |
| Flexible schedule | 1.49  (1.14, 1.95)** | 1.11  (0.65, 1.91) | 0.93  (0.69, 1.25) | 0.92  (0.44, 1.93) |
| Practice philosophy | 2.36  (1.77, 3.14)*** | 0.75  (0.39, 1.44) | 1.49  (1.03, 2.16)* | 0.91  (0.34, 2.43) |
| Pleasant patients | 0.96  (0.74, 1.25) | 1.28  (0.70, 2.33) | 0.90  (0.67, 1.22) | 0.94  (0.33, 2.66) |
| Safety is a priority | 1.23  (0.89, 1.71) | 1.55  (0.73, 3.31) | 0.95  (0.66, 1.37) | 1.56  (0.51, 4.77) |
| Helping patients | 1.14  (0.88, 1.47) |  | 1.27  (0.95, 1.68) |  |
| **Age (years) (reference: under 35)** | ***P<0.001*** | ***P = 0.92*** | ***P<0.001*** | ***P = 0.78*** |
| 35–44 | 1.46  (1.16, 1.82)*** | 0.91  (0.56, 1.43) | 1.13  (0.85, 1.51) | 0.89  (0.51, 1.57) |
| 45–54 | 1.74  (1.35, 2.23)*** | 1.05  (0.65, 1.71) | 1.86  (1.34, 2.56)*** | 0.83  (0.45, 1.56) |
| 55 and over | 2.36  (1.81, 3.09)*** | 1.04  (0.59, 1.83) | 2.12  (1.49, 3.00)*** | 0.69  (0.34,1.40) |
| **Race/Ethnicity (reference: Non-Hispanic, White)** | ***P = 0.30*** | ***P = 0.93*** | ***P = 0.97*** | ***P = 0.50*** |
| Non-Hispanic, Non-  White^1^ | 0.99  (0.75, 1.30) | 1.11  (0.66, 1.86) | 1.04  (0.76, 1.42) | 1.25  (0.72, 2.19) |
| Hispanic | 1.24  (0.94, 1.64) | 1.01  (0.59, 1.75) | 1.03  (0.75, 1.41) | 0.82  (0.44, 1.54) |
| **Employment Status (reference: Full-time)** | ***P = 0.44*** | ***P = 0.78*** | ***P = 0.36*** | ***P = 0.042*** |
| Part-time | 0.93  (0.77, 1.12) | 1.05  (0.74, 1.48) | 0.87  (0.63, 1.18) | 1.81  (1.02, 3.21)* |
| **Tenure (reference: 1–2 years)** | ***P = 0.058*** | ***P = 0.085*** | ***P = 0.054*** | ***P = 0.11*** |
| Less than 1 year | 1.03  (0.78, 1.36) | 1.00  (0.56, 1.81) | 1.38  (0.97, 1.95) | 0.97  (0.51, 1.83) |
| 3–5 years | 1.14  (0.90, 1.44) | 0.61  (0.38, 0.98)* | 0.87  (0.65, 1.18) | 1.17  (0.67, 2.03) |
| 6–10 years | 1.23  (0.93, 1.61) | 1.26  (0.76, 2.09) | 0.95  (0.67, 1.35) | 0.37  (0.15, 0.92)* |
| 11–20 years | 1.42  (1.07, 1.88)** | 1.32  (0.77, 2.25) | 1.26  (0.85, 1.88) | 1.46  (0.69, 3.10) |
| More than 20 years | 1.65  (1.16, 2.35)** | 0.76  (0.38, 1.58) | 1.44  (0.94, 2.21) | 0.62  (0.24, 1.60) |
| **Primary Practice Setting (reference: Private solo)** | ***P = 0.11*** | ***P = 0.060*** | ***P = 0.002*** | ***P = 0.40*** |
| Group | 0.94  (0.77, 1.15) | 0.65  (0.43, 0.99)* | 0.71  (0.54, 0.93)* | 0.83  (0.47, 1.46) |
| Specialty practice/Multi  specialty clinic | 1.05  (0.70, 1.57) | 1.36  (0.55, 3.32) | 1.09  (0.78, 1.52) | 0.69  (0.36, 1.33) |
| DSO^2^ | 1.12  (0.81, 1.54) | 1.32  (0.81, 2.16) | 0.54  (0.36, 0.82)** | 0.67  (0.32, 1.42) |
| Public health/FQHCs  /CHCs^3^ | 1.23  (0.85, 1.78) | 0.74  (0.33, 1.62) | 1.00  (0.65, 1.53) | 1.69  (0.75, 3.78) |
| Academic^4^ | 1.70  (1.14, 2.53)** | 1.78  (0.79, 4.03) | 1.34  (0.86, 2.10) | 0.81  (0.38, 1.74) |
| **Primary Practice Location (reference: Metropolitan)** | ***P = 0.58*** | ***P = 0.49*** | ***P = 0.38*** | ***P = 0.027*** |
| Micropolitan | 0.85  (0.63, 1.15) | 1.08  (0.60, 1.93) | 1.30  (0.89, 1.88) | 2.35  (1.15, 4.80)* |
| Small town/rural | 0.97  (0.67, 1.41) | 0.56  (0.21, 1.50) | 0.98  (0.64, 1.50) | 0.58  (0.21, 1.57) |
| **Geographic Region (reference: West)** | ***P = 0.55*** | ***P = 0.90*** | ***P = 0.65*** | ***P = 0.80*** |
| Northeast | 1.04  (0.80, 1.34) | 1.22  (0.73, 2.02) | 1.06  (0.77, 1.45) | 1.14  (0.61, 2.11) |
| Midwest | 1.02  (0.82, 1.28) | 1.11  (0.69, 1.79) | 0.99  (0.72, 1.35) | 0.97  (0.50, 1.91) |
| South | 1.16  (0.94, 1.44) | 1.07  (0.68, 1.67) | 0.87  (0.62, 1.21) | 1.29  (0.68, 2.42) |
| Constant | 0.26  (0.13, 0.50)*** | 0.43  (0.13, 1.44) | 0.76  (0.38, 1.5) | 0.34  (0.1, 1.13) |

Source: ADA Health Policy Institute. Dental Hygienists and Dental Assistants Workforce Shortage Survey, 2022.

Note: The dissatisfied group consisted of respondents who rated job satisfaction between 1 and 5 (very dissatisfied, 1–3; somewhat dissatisfied, 4–5) on a 10-point scale, whereas the satisfied group included those who rated it between 6 and 10 (somewhat satisfied, 6–7; very satisfied, 8–10).

^1^ Non-Hispanic, Non-White includes Black, American Indian, Alaska Native, Asian, Native Hawaiian, and Pacific Islander.

^2^ DSO, dental service organization.

^3^ FQHCs, Federally Qualified Health Centers; CHCs, Community Health Centers.

^4^ Academic includes university, college, school-based, military, independent, and other.

^5^ The Northeast region includes Connecticut, Maine, Massachusetts, New Hampshire, New Jersey, New York, Pennsylvania, Rhode Island, and Vermont. The Midwest region encompasses Illinois, Indiana, Iowa, Kansas, Michigan, Minnesota, Missouri, Nebraska, North Dakota, Ohio, South Dakota, and Wisconsin. The South region comprises Alabama, Arkansas, Delaware, District of Columbia, Florida, Georgia, Kentucky, Maryland, Mississippi, North Carolina, Oklahoma, South Carolina, Texas, Virginia, and West Virginia. The West region comprises Alaska, Arizona, California, Colorado, Hawaii, Idaho, Montana, Nevada, New Mexico, Oregon, Utah, Washington, and Wyoming.

**P*<0.05, ** *P*<0.01, and *** *P*<0.001
